# *Bartonella* spp. in Bats, Guatemala

**DOI:** 10.3201/eid1707.101867

**Published:** 2011-07

**Authors:** Ying Bai, Michael Kosoy, Sergio Recuenco, Danilo Alvarez, David Moran, Amy Turmelle, James Ellison, Daniel L. Garcia, Alejandra Estevez, Kim Lindblade, Charles Rupprecht

**Affiliations:** Author affiliations: Centers for Disease Control and Prevention, Fort Collins, Colorado, USA (Y. Bai, M. Kosoy);; Centers for Disease Control and Prevention, Atlanta, Georgia, USA (S. Recuenco, A. Turmelle, J. Ellison, C. Rupprecht);; Universidad del Valle de Guatemala, Guatemala City, Guatemala (D. Alvarez, D. Moran, A. Estevez);; Centers for Disease Control and Prevention Regional Office for Central America and Panama, Guatemala City (D.L. Garcia, K. Lindblade)

**Keywords:** Bartonella spp., bats, Desmodus rotundus, Guatemala, bacteria, dispatch

## Abstract

To better understand the role of bats as reservoirs of *Bartonella* spp., we estimated *Bartonella* spp. prevalence and genetic diversity in bats in Guatemala during 2009. We found prevalence of 33% and identified 21 genetic variants of 13 phylogroups. Vampire bat–associated *Bartonella* spp. may cause undiagnosed illnesses in humans.

Multiple studies have indicated that bats might serve as natural reservoirs to a variety of pathogens, including rabies virus and related lyssaviruses, Nipah and Hendra viruses, Marburg virus, and others (*1*,*2*). Bats’ high mobility, broad distribution, social behavior (communal roosting, fission–fusion social structure), and longevity make them ideal reservoir hosts and sources of infection for various etiologic agents. In addition to viruses, bacteria and ectoparasites have been detected in bats (*3*–*5*) and can potentially cause human infection (*6*).

*Bartonella* spp. have been found in rodents, insectivores, carnivores, ungulates, and many other mammals. Naturally infected hematophagous arthropods, such as fleas, flies, lice, mites, and ticks are frequently implicated in transmitting *Bartonella* spp (*3*–*5*,*7*). Detection of *Bartonella* DNA in the saliva of dogs suggests the possibility that *Bartonella* spp. can be transmitted through biting (*8*). Increasing numbers of *Bartonella* spp. have been identified as human pathogens (*9*,*10*). However, a mammalian reservoir has not been determined for some newly identified species, such as *B. tamiae* (*9*). Extensive surveillance for *Bartonella* spp. among diverse groups of animals, including bats, has become crucial.

To our knowledge, *Bartonella* spp. in bats have been studied only in the United Kingdom and Kenya (*11*,*12*). To better understand the role of bats as reservoir hosts of *Bartonella* spp. and their potential risk for infecting humans and animals, we looked for *Bartonella* spp. in bats in Guatemala, estimated prevalence, and evaluated the genetic diversity of the circulating *Bartonella* strains.

## The Study

**Figure 1:**
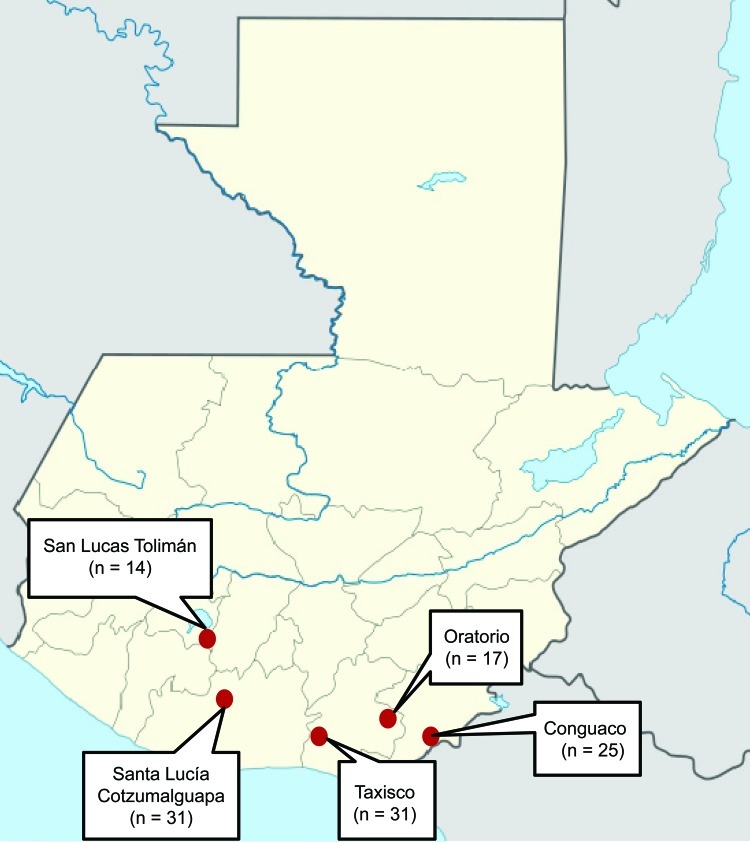
Sites of bat collection, showing number of bats collected from each site, Guatemala, 2009.

In 2009, a total of 118 bats were collected from 5 sites in southern Guatemala (Figure 1). The bats represented 15 species of 10 genera; the most prevalent (26.3%) species was the common vampire bat (*Desmodus rotundus*); the other 14 species accounted for 0.8%–12.7% of the bats sampled. Diversity of bats was 6–8 species per site (Table 1). Blood specimens from the bats were collected and cultured for *Bartonella* spp., according to a published method (*12*). A total of 41 *Bartonella* isolates were obtained from 39 (33.1%) of the 118 bats; colonies with different morphologic characteristics were identified from blood of 2 *Pteronotus davyi* bats. Prevalence of *Bartonella* spp. in Conguaco (60%, 15/25) was significantly higher than that in Oratorio (11.8%, 2/17), San Lucas Tolimán (14.3%, 2/14), and Taxisco (22.6%, 7/31) but did not differ from that in Santa Lucía Cotzumalguapa (41.9%, 13/31). *Bartonella* spp. were cultured from 8 bat species. The *Bartonella* spp. prevalence among *Phyllostomus discolor* (88.8%, 9/8), *P. davyi* (70%, 7/10), and *D. rotundus* (48.4%, 15/31) bats was significantly higher than that among *Sturnira lilium* (8.3%, 1/12) and *Glossophaga soricina* (13.3%, 2/15) bats. No *Bartonella* spp. were found in *Artibeus jamaicensis* (0/13) and 6 other bat species tested (Table 1).

**Table 1:** Prevalence of *Bartonella* spp. in bats from 5 collection sites, Guatemala, 2009

Bat species	No. positive/no. cultured					Overall, no. positive/ no. cultured (%)
	Conguaco	Oratorio	San Lucas Tolimán	Santa Lucía Cotzumalguapa	Taxisco	
*Artibeus jamaicensis*	0/3	0/2	0	0/3	0/5	0/13
*Artibeus lituratus*	0/1	0/1	0/1	0	0	0/3
*Artibeus toltecus*	1/1	0	0	0	0	1/1 (100)
*Carollia castanea*	0	0	0	0/1	0	0/1
*Carollia perspicillata*	0	0/2	0/3	0	4/9	4/14 (28.6)
*Desmodus rotundus*	5/7	2/4	0/1	6/12	2/7	15/31 (48.4)
*Glossophaga soricina*	1/1	0/3	1/5	0/3	0/3	2/152 (13.3)
*Micronycteris microtis*	0	0	0	0	1/3	1/3 (33.3)
*Myotis elegans*	0	0	0	0/1	0/1	0/2
*Myotis nigricans*	0	0	0	0	0/1	0/1
*Phyllostomus discolor*	7/8	0	1/1	0	0	8/9 (88.9)
*Platyrrhinus helleri*	0	0	0	0/1	0	0/1
*Pteronotus davyi*	0	0	0	7/10	0	7/10 (70)
*Sturnira lilium*	1/3	0/5	0/2	0	0/2	1/12 (8.3)
*Sturnira ludovici*	0/1	0	0/1	0	0	0/2
Total	15/25	2/17	2/14	13/31	7/31	39/118 (33.1)

**Figure 2:**
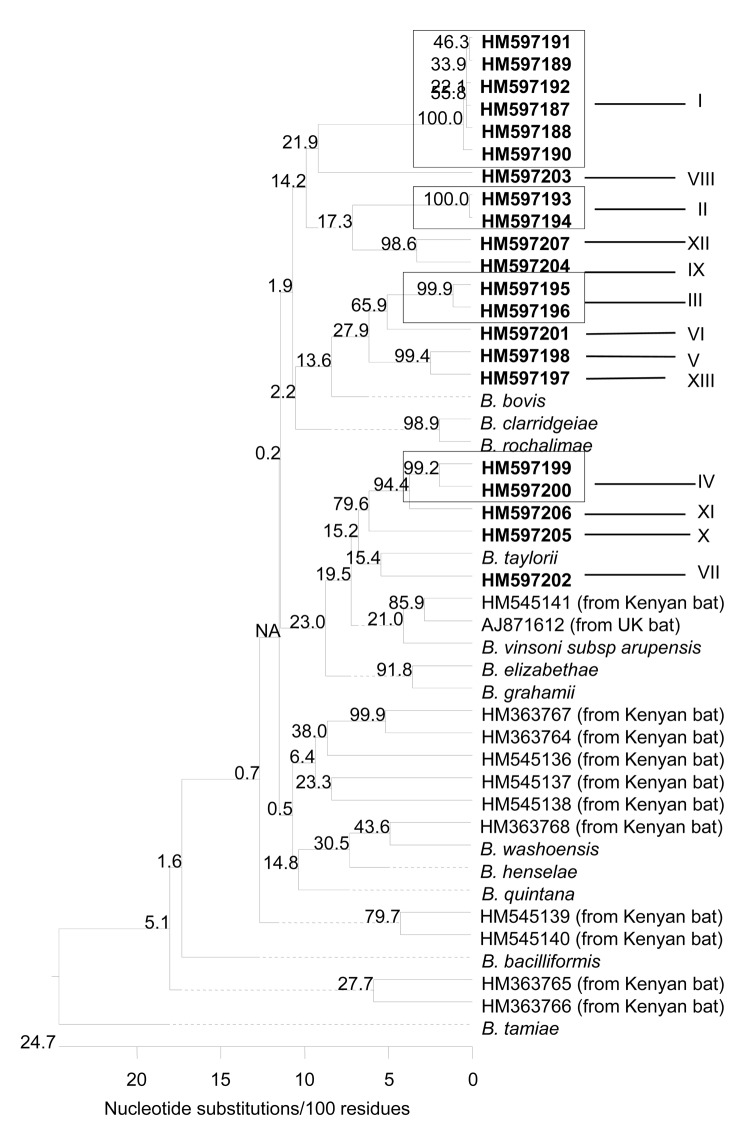
Phylogenetic relationships of the *Bartonella* spp. genotypes based on partial sequences of the citrate synthase gene detected in bats from Guatemala, Kenya, United Kingdom, and some reference *Bartonella* spp. The phylogenetic tree was constructed by the neighbor-joining method, and bootstrap values were calculated with 1,000 replicates. A total of 21 *Bartonella* genotypes, forming 13 *Bartonella* phylogroups, were identified in the bats from Guatemala. Each genotype is indicated by its GenBank accession number in **boldface**; the phylogroups are marked by Roman numerals I–XIII.

Identity of 41 *Bartonella* isolates was confirmed by PCR amplification of a specific region in the citrate synthase gene by using primers BhCS781.p (5′-GGGGACCAGCTCATGGTGG-3′) and BhCS1137.n (5′-AATGCAAAAAGAACAGTAAACA-3′). Subsequent sequencing analyses of the 41 isolates revealed 21 genetic variants (Table 2) that clustered into 13 phylogroups (I–XIII) with 6.6%–24.7% divergence. The phylogroups were also distant from any previously described *Bartonella* species and genotypes identified in bats from the United Kingdom and Kenya (Figure 2). Each phylogroup contained 1–6 variants; similarities within phylogroups were 96.2%–99.7% (Table 2).

**Table 2:** GenBank accession numbers and distribution of 21 genetic variants of *Bartonella* spp. in bats from Guatemala, 2009

Accession no.	Type strain	Host bat species	No. sequences	Distribution (no. isolates)	Phylogroup
HM597187	B29042	*Desmodus rotundus*	1	*D. rotundus* (1)	I
HM597188	B29043	*D . rotundus*	3	*D. rotundus* (3)	I
HM597189	B29044	*D . rotundus*	2	*D. rotundus* (2)	I
HM597190	B29107	*D . rotundus*	1	*D. rotundus* (1)	I
HM597191	B29108	*D. rotundus*	3	*D. rotundus* (2); *C. perspicillata* (1)	I
HM597192	B29114	*D. rotundus*	3	*D. rotundus* (2); *C. perspicillata* (1)	I
HM597193	B29102	*Pteronotus davyi*	3	*P. davyi* (3)	II
HM597194	B29109	*P. davyi*	1	*P. davyi* (1)	II
HM597195	B29119	*D. rotundus*	3	*D. rotundus* (3)	III
HM597196	B29122	*D. rotundus*	1	*D. rotundus* (1)	III
HM597198	B29116	*Phyllostomus discolor*	2	*P. discolor* (2)	V
HM597199	B29126	*Carollia perspicillata*	2	*C. perspicillata* (2)	IV
HM597200	B29230	*P. discolor*	1	*P. discolor* (1)	IV
HM597201	B29115	*P. discolor*	3	*P. discolor* (3)	VI
HM597202	B29110	*Glossophaga soricina*	3	*G. soricina* (2); *P. davyi* (1)	VII
HM597203	B29105	*P. davyi*	3	*P. davyi* (3)	VIII
HM597204	B29112	*P. discolor*	2	*P. discolor* (2)	IX
HM597205	B29134	*P. davyi*	1	*P. davyi* (1)	X
HM597206	B29137	*Sturnira lilium*	1	*S. lilium* (1)	XI
HM597207	B29172	*Micronycteris microtis*	1	*M. microtis* (1)	XII
HM597197	B29111	*Artibeus toltecus*	1	*A. toltecus* (1)	XIII

Of the 13 phylogroups, phylogroups I, IV, and VII were identified in isolates obtained from different bat species (Table 2), suggesting that bats of different species may share the same *Bartonella* strain; whereas 4 species of bats—*C. perspicillata*, *D. rotundus*, *P. discolor*, and *P. davyi*—were infected with 2–4 *Bartonella* strains (Table 2). *P. davyi* from 2 bats belonged to phylogroups II or VIII.

## Conclusions

The high (≈33%) prevalence of *Bartonella* spp. in bat populations in southern Guatemala might suggest persistent infection of long-lived bats with *Bartonella* spp., similar to their infection with some viruses (*13*). Depending on the bat species, *Bartonella* spp. exhibit high, low, or no infectivity, which may explain the variation in *Bartonella* spp. prevalence between study sites because the assemblage of bat species differed at each site. Additional studies are needed to illustrate the distribution of *Bartonella* spp. among the bat fauna in Guatemala and throughout the region.

Further characterization is necessary to verify whether the *Bartonella* strains representing a variety of distinct phylogroups represent novel *Bartonella* species. Unlike the discovery in bats in Kenya (*12*), host specificity of *Bartonella* spp. was not found in bats in Guatemala. Such lack of specificity may be partly associated with the arthropod vectors that parasitize bats, although we were unable to attempt isolation of agents from the bat ectoparasites. Future studies of bat ectoparasites would enable testing of hypotheses about whether any arthropods may be vectors in the *Bartonella* spp. transmission cycle and whether ectoparasite specificity contributes to the lack of host specificity observed in this study.

The tendency of some bat species to share roosts, reach large population densities, and roost crowded together creates the potential for dynamic intraspecies and interspecies transmission of infections (*14*). In accordance with this hypothesis, our finding that co-infection with multiple *Bartonella* strains in a single bat species, and even in an individual bat, indicate that active interspecies transmission of *Bartonella* spp. likely occurs among bats in Guatemala. The specificity of ectoparasite arthropod vectors among the bat fauna remains unclear and may contribute to interspecies transmission of *Bartonella* spp. among bats.

The long life spans of bats (average 10–20 years) may have made them major reservoirs that contribute to the maintenance and transmission of *Bartonella* spp. to other animals and humans. The bite of the common vampire bat has been long recognized to transmit rabies virus to humans throughout Latin America (*2*). These bats typically feed on the blood of mammals, including domestic animals and humans (*15*). Predation of vampire bats on humans is a major problem in Latin America (*2*). If *Bartonella* spp. can be transmitted to humans through the bite of bats, the need for further studies with vampire bats is imperative. *Bartonella* spp.–specific DNA has been detected in ectoparasites collected from bats (*3*–*5*). Presumably, if *Bartonella* spp. are transmitted through a bat ectoparasite vector, some, if not all, bat-associated *Bartonella* spp. could be transmitted to humans because bats are frequent hosts to a wide variety of ectoparasites, including bat flies, fleas, soft ticks, and mites. However, transmission potential might vary with the degree of synanthropic roosting or foraging behavior within the bat community.

Because an increasing number of *Bartonella* spp. are being associated with human illness, the need to identify the animal reservoirs of these novel *Bartonella* spp. and to understand their disease ecology is also increasing. Our study of *Bartonella* spp. in bats has enlarged our scope of this zoonotic potential as we search for the reservoirs that harbor novel and known *Bartonella* spp.
